# Breast cancer mortality in Saudi Arabia: Modelling observed and unobserved factors

**DOI:** 10.1371/journal.pone.0206148

**Published:** 2018-10-22

**Authors:** Refah Mohammed Alotaibi, Hoda Ragab Rezk, Consul Iworikumo Juliana, Chris Guure

**Affiliations:** 1 Department of Mathematical Sciences, Faculty of Science, Princess Nourah bint Abdulrahman University, Riyadh, Saudi Arabia; 2 Department of statistics, Al-Azhar University, Cairo, Egypt; 3 Department of Mathematics, Computer Science, Niger Delta University, Bayelsa State, Nigeria; 4 Department of Biostatistics, School of Public Health, University of Ghana, Legon, Ghana; University of South Alabama Mitchell Cancer Institute, UNITED STATES

## Abstract

**Background:**

Breast cancer is one of the most dangerous and frequently occurring cancers among women, and it also affects men. We aimed to determine the prevalence and factors associated with mortality among patients with breast cancer in Saudi Arabia.

**Method:**

Data for this analysis of breast cancer mortality among Saudi Arabians were obtained from the Saudi Arabian Cancer Registry at the King Faisal Hospital and Research Centre. Both descriptive and inferential statistical analyses were conducted using proportions, chi-squared tests, and the Cox regression model. Frequentist and Bayesian inferential statistics were used to estimate the risk ratios. A frailty term was specified to control for suspected heterogeneity across regions. Bayesian and deviance information criteria were used to discriminate between the frequentist and Bayesian frailty models, respectively.

**Results:**

Out of 5,411 patients, 708 (13.08%) deaths occurred that were attributable to breast cancer. Of those, 12 (1.69%) were men. Among patients who died of breast cancer, 353 (49.86%) had tumours that originated on the left side and 338 (47.74%) on the right side. In terms of the stage or extent of breast cancer, 318 (44.92%) deaths occurred among patients who had distant metastases, followed by 304 (42.94%) who had regional metastases and 86 (12.15%) with localized cancers. Men were 72% more likely than women to die from breast cancer. Divorcees were twice as likely to die, compared to their married counterparts. Patients whose tumours were classified as Grade IV had the highest mortality rate, which was 5.0 times higher than patients with Grade I tumours (credible interval (CrI); 1.577, 14.085) and 3.7 times higher than patients with Grade II tumours (CrI; 1.205, 9.434).

**Conclusion:**

There is a high prevalence of breast cancer mortality among Saudi Arabian women, with the highest prevalence among divorced women. Though the prevalence of breast cancer mortality among men is lower than that of women, men had a higher risk of death. We therefore recommend an intensive health education programme for both men and women. These programmes should discuss the consequences of divorce, the prevalence of breast cancer among men, and early diagnoses and treatments for breast cancer.

## Introduction

Breast cancer is one of the most dangerous and frequently occurring cancers among women. Breast cancer starts when cells in the breasts grow out of control and form a tumour. Signs of breast cancer include a lump in the breast, a change in breast size, pain in the breast, and fluid discharge from the nipple. The most common symptom of breast cancer in men is a hard, painless lump in one of the breasts. According to the American Cancer Society [1], factors that affect one’s chances of developing breast cancer include age, lifestyle (e.g., alcohol consumption), and family history. Factors affecting the mortality of breast cancer patients include education level [2] and race Bartonsville [3]. Mar et al. [4] assessed breast cancer rates based on age and other risk factors, such as family history and genetic factors. Bernd et al. [5] and Mar et al. [4] discuss how mammography screening can reduce mortality rates in breast cancer. [5] also assess mortality trends in breast cancer by age and stage.

Breast cancer is a common malignancy among Saudi females, with a prevalence of 21.8%. The most recent survey of cancer-related mortality among Saudi women finds that breast cancer is the ninth leading cause of death [6, 7, 8]. Al-Qahtani [9] reports that breast cancer is the second most common malignancy in Saudi women. Ibrahim et al. [10] predict that breast cancer rates in Saudi Arabia will increase over the next few decades as the population grows and ages. According to the Saudi Cancer Registry of the King Faisal Specialist Hospital and Research Centre, around 930 new cases of breast cancer are diagnosed each year in Saudi Arabia. In 2010, out of 5,378 cancer diagnoses in Saudi Arabia, 1,473 (27.4%) were for breast cancer, making it the most common newly diagnosed cancer among women.

Anders et al. [11] find that in the US, approximately 7% of women with breast cancer are diagnosed before age 40, and survival rates for these women are worse than for those diagnosed at older ages. Early diagnosis is an important issue among young Saudi women. The 2002 annual report of Saudi National Cancer Registry shows that breast cancers that develop before age 40 comprise 26.4% of all female breast cancers in Saudi Arabia, compared with 6.5% in the United States.

Mortality associated with breast cancer may depend on some unobserved or unknown covariates or risk factors, called frailties. This research considers the Bayesian and frequentist models of unobserved and observed factors that affect the mortality of patients with breast cancer. This research uses both the frequentist Cox proportional hazards model, with and without frailty, and the Bayesian Cox proportional hazards model, with and without frailty. We apply these models to a dataset of patients with breast cancer in Saudi Arabia to determine the factors associated with breast cancer mortality.

### Ethics statement

The 2012 Saudi Arabia Cancer Incidence Report details eligibility requirements for breast cancer. Patient eligibility was determined by a medical oncologist and by clinical, histopathological, and radiological diagnoses. Eligible patients at the hospitals were assigned to one of two groups for cause of death: breast cancer or other. The local review boards of the participating institutions in accordance with the Saudi Cancer Registry approved the treatment protocol. The institutional review board of the Saudi Cancer Registry and the local ethics committee of the Cancer Registry in King Faisal Specialist Hospital filed and approved the assurances. The primary site (topography) and histology (morphology) of the breast cancer malignancies were identified and coded according to the International Classification of Diseases for Oncology, 3rd edition (World Health Organization, 2000). Written informed consent and approval were obtained by the Saudi Cancer Registry. Data that were obtained and used in our analysis were all de-identified.

## Source of data and research variables

The Saudi Cancer Registry (SCR) of the King Faisal Specialist Hospital and Research Centre provided the data set. SCR is a national cancer registry of the Saudi Health Council. Established in 1992 under the authority of the Ministry of Health. SCR collects all data related to cancer registration from all 13 administrative regions in the Kingdom: Riyadh, Makkah, Madinah, Qassim, Hail, Jouf, Tabouk, Najran, Baha, Asir, Jezan, International and the eastern and northern regions. The SCR’s main office indirectly supervises the regional offices and ensures the accuracy and quality of the data.

The data set contains information on 8,312 patients with cancer, including 8,172 females (98%) and 140 males (1.68%) who were diagnosed with advanced breast cancer with some covariates. The data were collected for 9 years, from 2004 to 2013. Information includes survival time, censoring indicator, sex, age, marital status, demographics (e.g., address, nationality), and tumour details (e.g., laterality, site, behaviour, grade, stage, topography). The primary site (topography) and histology (morphology) of the malignancies are identified and coded according to the International Classification of Diseases for Oncology 3rd Edition (WHO, 2000). The data were entered in the computer using CanReg 4 (IACR) software (Cancer Incidence Report, 2010). Participants who did not provide responses for all study variables were deleted, eliminating 2,901 patients records. The remaining 5,411 patient records were used for the data analysis.

## Outcome variable

Breast cancer mortality occurs mainly because of cancer in the breast(s). In these data, we defined the outcome variable as patients who died from breast cancer. Specifically, our outcome variable was survival time in years for patients diagnosed with breast cancer. Patients who died from breast cancer were deemed to have had the event and assigned the number 1. Those who dropped out of the study, did not die within the period, or died from other diseases were censored and assigned the number 0.

## Explanatory variables

All explanatory variables included in this study are those that were obtained from patients during the time of the study. The description of variables in the data set are given as: **Age**: This variable provides the patient age at diagnosis. **Gender**: It refers to patient’s gender with the value ″1‶for male and ″2‶for female. **Grade**: The grade of a tumor describes how abnormal the tumor cell and tissue look under a microscope. It indicates how quickly a tumor can grow and spread. The tumour is well-differentiated as if the tumour cells and the organization of the tumor’s tissue are close to those of normal cells. These tumors tend to grow and spread at a slower rate. The undifferentiated or poorly differentiated tumors have abnormal-looking cells and may lack normal tissue structures. In our data set, we have used the value ″1‶for Grade I (well differentiated or low grade), the value ″2‶for Grade II (moderately differentiated or intermediate grade), the value ″3‶for Grade III (Poorly differentiated or high grade) and the value ″4‶for Grade IV (undifferentiated or high grade). **Stage or Extent**: This variable groups the breast cancer cases into broad categories based on the extent of disease. We have used the value ″1‶for Distant Metastasis, ″2‶for localised, ″3‶for regional. **Laterality**: This variable identifies the side of a paired organ or of the body on which the tumor originated. In our data we use the value ″1‶as a ″Bilateral Involve‶, ″2‶for ″Left‶, ″3‶for ″Paired site‶and ″4‶for ″right‶. **Topography**: The variable indicates the site of origin of the tumor or where the tumor arose. The breast halves are divided into quarters or quadrants. The *ICD* − *O* − 3 code for upper-inner quadrant is *C*50.2, lower-inner quadrant is *C*50.3, upper-outer quadrant is *C*50.4, and lower-outer quadrant is *C*50.5. In our data set, we have used the value ″1‶for ″nipple‶, ″2‶for ″C50.1 Central portion of breast‶, ″3‶for ″Upper-inner quadrant of breast‶, ″4‶for ″Lower-inner quadrant of breast‶, ″5‶for ″Upper-outer quadrant of breast‶, ″6‶for ″Lower-outer quadrant of breast‶, ″7‶for ″Axillary tail of breast‶, ″8‶for ″Overlapping lesion of breast‶and ″9‶for ″Breast, NOS‶. **Marital status**: In the data set, we have used the value ″1‶for ″divorced‶, ″2‶for ″married‶, ″3‶for ″single‶and ″4‶for widowed. **Address code**: We have used the value ″1‶for ″Eastern‶, ″2‶for ″Riyadh‶, ″3‶for ″Asir‶‶, ″4‶for ″Tabuk‶, ″5‶for ″Qassim‶, ″6‶for ″Madinah‶, ″7‶‶for ″Makkah‶, ″8‶for ″Hail‶, ″9‶for ″Jouf‶, ″10‶for ″Baha‶, ″11‶for ″Northern‶, ″12‶for ″Jazan‶, ″13‶for ″International‶and ″14‶for ″Najran‶.

## Analytical approach

Bayesian and frequentist methodologies were implemented in this study. Four different models were specified, with and without a frailty term. The models included 1) the frequentist standard Cox proportional hazards model, 2) the frequentist standard Cox proportional hazards model with frailty, 3) the fully Bayesian Cox proportional hazards model, and 4) the fully Bayesian Cox proportional hazards model with frailty. We assumed that all participants were independent of each other, irrespective of regional distribution of patients, hence models 1 and 3. Models 2 and 4 were used to indicate patients’ regions of residence, as the likelihood of similar traits among patients from the same region was not necessarily independent. These models allowed us to analyse heterogeneity across regions.

### The Cox proportional hazards model with and without frailty term

A Cox proportional hazards model is a statistical technique for exploring the relationship between patient survival and several covariates. It estimates the treatment effect on survival after adjusting for other covariates. It also estimates the hazard (or risk) of death for an individual, given the prognostic variables. Cox (1972) proposed using proportional hazards in medical testing analysis and modelling the effect of secondary variables on survival. The hazard function depends on the covariates, which may be either independent or dependent. The Cox proportional hazards model without frailty assumes that individual survival times are independent of each other. Appendix 1 describes the mathematical expressions of the Cox model without frailty, as specified and used in the paper.

The Cox proportional hazards model without frailty assumes that individual survival times are independent. However, survival-related factors may have within-group commonalities, such as siblings or households. The frailty model can model these within-group associations, including individual survival times within groups. The frailty is an unobserved random effects variable that is shared by subjects within the group. A frailty acts multiplicatively on the hazard ratio of all group members. With this model, groups with a large frailty value experience the event at an earlier stage than groups with small frailty values. Appendix 2 provides further details of the Cox model with frailty.

### Bayesian proportional hazards model with and without frailty term

The Bayesian inference employs Bayes’ theorem, which can be used to show the relationship between two conditional probabilities. Bayes’ theorem combines prior experience (i.e., prior probability) with observed data (i.e., likelihood) to interpret the data (i.e., posterior distribution). A semi-parametric approach to specifying the hazard of a model often is preferable to a fully parametric model, As in Sinha and Dey [12], a semi-parametric approach to specifying the hazard of a model is preferable to a fully parametric model, because the former avoids specifying the time dependence parametrically and hence mis-specifying the parametric form. Appendix 3 details the mathematical expression of both the with and without Bayesian frailty approach.

## Data analysis

Analysis of the data was conducted using Stata, R, and INLA software packages. Stata was used for data cleaning, descriptive analysis, and testing the proportional hazards assumptions. R was used for the frequentist approaches, using Bayesian information criteria to discriminate between the *with* and *without* frailty models. The INLA software was used for the Bayesian approaches, with and without frailty. The with-frailty and without-frailty approaches enabled us to determine whether models with frailty were best for our data. For the Bayesian approaches, the deviance information criterion was used. Libraries such as *Mass*, *Survival*, and *frailty* were used for data analysis. Bayesian estimates were used to interpret the results.

### Test of proportionality under survival analysis

To test for the proportional hazards assumption under the standard Cox model, we conducted the Schoenfeld residual test and a graphical approach. The Schoenfeld test hypothesises that some variables do not vary with time. This hypothesis implies that some variables remained constant over the study period and therefore satisfied the proportionality assumption under the standard Cox model. We stratified variables that did not satisfy this condition but were significant. We used four models for this analysis: the frequentist-stratified Cox proportional hazards *with* and *without* a frailty term and the Bayesian-stratified Cox proportional hazards *with* and *without* a frailty term. We specified ‘region of the country’ as the frailty term in all models.

## Results

### Descriptive analysis

Table 1 shows the distribution of events (patients who died from breast cancer) for each of the factor variables. For the continuous variable (age), we obtained the mean (standard deviation). Out of 5,411 deaths, 708 (13.08%) were attributable to breast cancer. As shown in the Table 1, only 12 (1.69%) male patients died from breast cancer. Among married patients, 582 (82.20%) died from breast cancer, followed by 62 (8.7%) deaths among widows. Among single patients, 37 (5.23%) died from breast cancer, and 27 (3.8%) deaths among divorced patients. Among patients who died from breast cancer, 353 (49.86%) had tumours that originated on the left side and 338 (47.74%) on the right side. In terms of the stage or extent of disease, 318 (44.92%) patients who died were categorized as ″distant metastasis‶, followed by 304 (42.94%) whose cancers were categorized as regional and 86 (12.15%) as localized.

**Table 1:** Frequency distribution of cancer deaths according to the independent variables.

variable	Freq(%)	variable	Freq(%)
**Gender**		**Extent**	
Female	696(98.31)	Distant Metastasis	318(44.92)
Male	12(1.69)	Localised	86(12.15)
		Regional	304(42.94)
**Marital status**		**Laterality**	
Divorced	27(3.81)	Bilateral Involve	12(1.69)
Married	582(82.20)	Left	353(49.86)
Single	37(5.23)	Paired site, late	5(0.71)
Widowed	62(8.76)	Right	338(47.74)
**Topography**		**Grade**	
Nipple	28(3.95)	Grade I (Well diff)	26(3.67)
Central portion of breast	22(3.11)	Grade II (Mod diff)	277(39.12)
Upper-inner quadrant of breast	36(5.08)	Grade III (Poor diff)	382(53.95)
Lower-inner quadrant of breast	24(3.39)	Grade IV (Undiff Anaplastic)	23(3.24)
Upper-outer quadrant of breast	142(20.06)		
Lower-outer quadrant of breast	22(3.11)		
Axillary tail of breast	4(0.56)		
Overl. lesion of breast	139(19.63)		
Breast, NOS	291(41.10)	**Age**	48.48 (12.59)

Of the 5,411 patients in the dataset, 1,528 were from the capital of Saudi Arabia, Riyadh, followed by 1,432 (26.46%) from Makkah and 1,101 (20.35%) from the eastern region, with only 9 (0.17%) from the international region. Riyadh accounted for 280 breast cancer deaths, including 129 categorized as distant metastasis and 19 as regional. Similarly, out of 146 deaths in Makkah, 53 were classified as distant metastasis and 66 as regional. Out of 98 deaths in the eastern region, 34 were classified as distant metastasis and 59 as regional.

### Bivariate analysis

We sought to establish whether breast cancer stage (distant metastasis, localised, or regional) could be categorised according to the region in which the patient lived. We found a statistically significant difference across the region of residence after applying the Pearson’s chi-squared test statistic. The chi-squared test statistic was 81.10, with a corresponding p-value <.001. We also assessed the cancer grade, where Grade I is well differentiated or low grade, Grade II is moderately differentiated or intermediate grade, Grade III is poorly differentiated or high grade, and Grade IV (undifferentiated or high grade) also showed a significant difference across the region of residence.

### Testing the proportional hazards assumption

**Fig 1:**
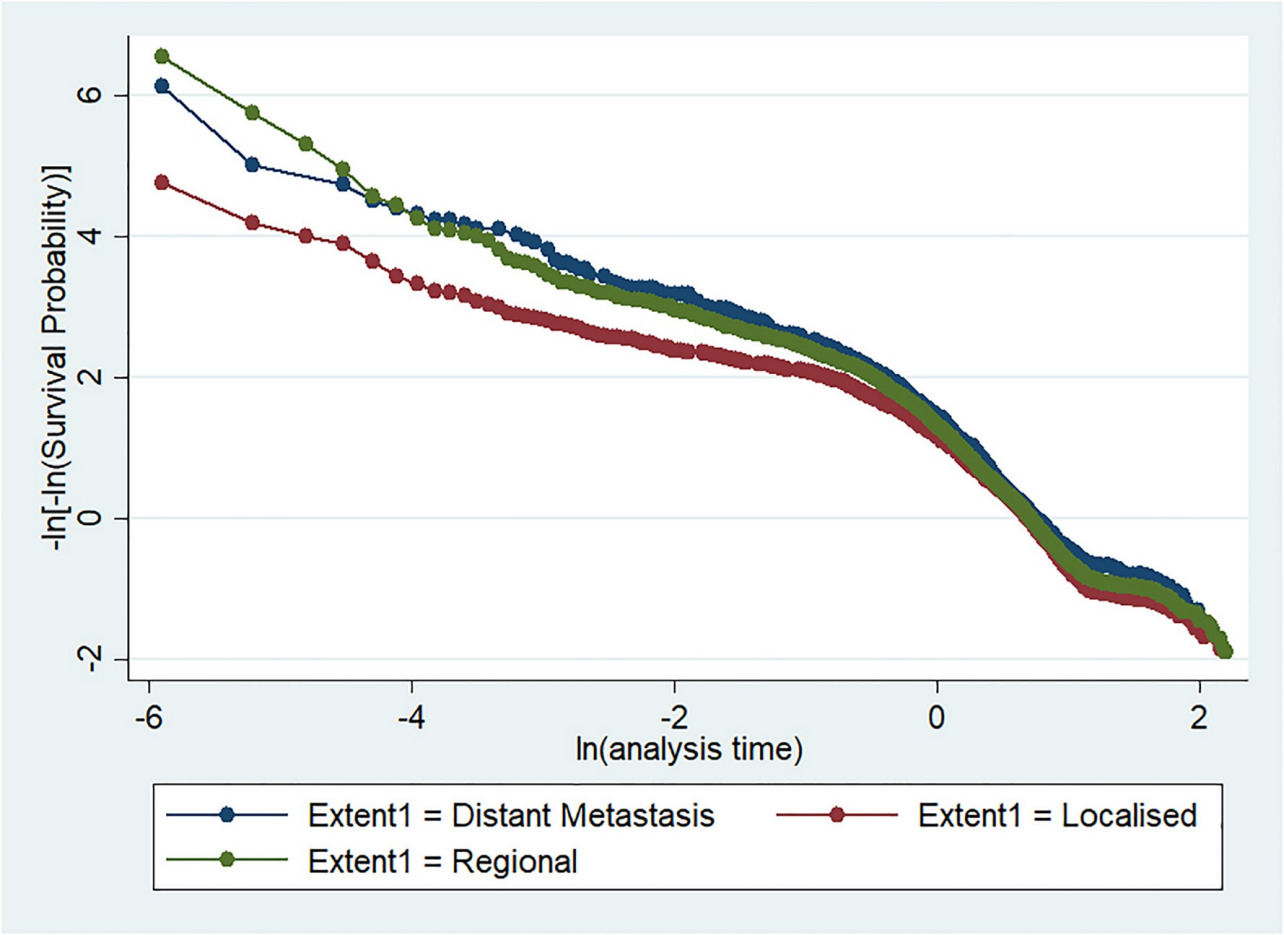
Graphical representation of the Cox proportional hazards assumption with the extent or stage variable.

Table 2 presents the results for testing the proportionality hazards assumption for each variable and the global text. All variables, except disease stage or extent, met the standard for the Cox proportional hazards model. Because the extent variable was significant and because it violated the proportional hazards assumption, it could not be dropped or removed from the analysis. Fig 1 graphs the variable (stage/extent) that did not satisfy the proportional hazards assumption. Thus, to control for the extent variable, we used a stratification approach.

**Table 2:** Test of the Cox proportional hazards regression model assumption.

Time:Time		
	Chi2	prob > chi2
Gender2	0.41	0.521
Age	0.02	0.880
Marital sta2	3.01	0.083
Topography	1.00	0.317
Grade	0.38	0.539
Laterality	1.28	0.257
CauseDeath	2.77	0.096
Extents	0.65	0.010
Global test	15.27	0.044

### Multivariate (adjusted-risk ratios) analysis

Table 3 presents the factors associated with breast cancer mortality that were obtained using the Bayesian approach. The deviance information criteria for the stratified Cox model with and without frailty were 6852.70 and 6887.62, respectively. These values indicate that the frailty model was a better fit than the model without frailty. From this model, men were about 72% more likely to die from breast cancer, compared to their female counterparts, though it was not statistically significant. Age was a higher and statistically more significant predictor of mortality. Our results indicate that, for every unit increase in age, there was a corresponding 0.7% increase in mortality. We also observed that patients who reported having tumours originating from a paired site and from the left side were at higher risk of mortality, compared to bilateral tumour involvement.

**Table 3:** Bayesian hazards ratio with their corresponding credible intervals using the Cox proportional hazards regression model.

	Without frailty		With frailty	
	HR	95%Crl	HR	95%Crl
**Gender**				
Female	Ref			
Gender2Male	1.720	(0.920, 2.979)	1.718	(0.916, 2.981)
**Marital Status**				
Divorced	Ref			
Married	0.459	(0.316, 0.690)	0.464	(0.319, 0.699)
Single	0.394	(0.238, 0.659)	0.422	(0.254, 0.706)
Widowed	0.473	(0.303, 0.755)	0.472	(0.302, 0.755)
**Age**	1.006	(1.000, 1.013)	1.007	(1.001, 1.013)
**Grade**				
Undiff Anaplastic	Ref			
Grade I (Well diff)	0.190	(0.070, 0.619)	0.194	(0.071, 0.634)
GradeGrade II (Mod diff)	0.258	(0.102, 0.789)	0.270	(0.106, 0.830)
GradeGrade III (Poor diff)	0.421	(0.167, 1.284)	0.445	(0.176, 1.368)
**Topography**				
Nipple	Ref			
Central portion of breast	0.539	(0.305, 0.941)	0.568	(0.321, 0.994)
Upper-inner quadrant of breast	0.797	(0.484, 1.321)	0.827	(0.501, 1.376)
Lower-inner quadrant of breast	0.844	(0.486, 1.457)	0.872	(0.501, 1.510)
Upper-outer quadrant of breast	0.650	(0.437, 0.996)	0.656	(0.440, 1.008)
Lower-outer quadrant of breast	0.440	(0.249, 0.770)	0.457	(0.258, 0.800)
Axillary tail of breast	0.674	(0.211, 1.788)	0.352	(1.508, 0.621)
Overl. lesion of breast	0.733	(0.492, 1.124)	0.747	(0.500, 1.150)
Breast, NOS	0.955	(0.655, 1.439)	1.014	(0.693, 1.534)
**Laterality**				
Bilateral Involve	Ref			
Left	1.027	(0.594, 1.918)	1.081	(0.567, 1.831)
Paired site	3.925	(1.220, 11.383)	3.269	(1.006, 9.597)
Right	0.983	(0.568, 1.835)	0.949	(0.549, 1.771)

Divorced patients were 2.2, 2.4, and 2.1 times more likely to die via breast cancer when compared to married, single, and widowed patients, respectively. These results all were statistically significant. Patients whose tumours were diagnosed and classified as Grade IV (undiff anaplastic) had the highest mortality rate. Patients with Grade IV cancers were 5 times more likely to die than those with Grade I cancers (credible interval (CrI): 1.577, 14.085). Patients with Grade IV cancers were 3.7 times more likely to die than those with Grade II cancers (CrI: 1.205, 9.434). All results were statistically significant. Also, patients with Grade IV cancers were 2.2 more likely to die than those with Grade III (CrI: 0.731, 5.682), though this difference was not statistically significantly different.

With reference to the topography variable, only two sites of the origin of the tumour were statistically significantly different from tumours that developed around the nipple. Patients who developed breast tumours that were not otherwise specified were 1.4% more at risk of death, compared to those with tumours at the nipple. Those whose tumours were located at the nipple were 1.761 (CrI; 1.006, 3.115) and 2.2 (CrI: 1.25, 3.88) times more likely to die than those with tumours in the central or lower outer quadrant of the breast, respectively (results were statistically significant). The results obtained using the frequentist frailty model presented in Table 4 were similar to those of the Bayesian approach. We dropped men from the sample to analyse factors associated with women’s higher prevalence of breast cancer mortality. Among women in the sample who had breast cancer, nearly all (98.31%) deaths in women were attributable to breast cancer.

**Table 4:** Frequentist hazards ratio with their corresponding confidence intervals using the Cox proportional hazards regression model.

	Without frailty		With frailty	
	HR	95%Cl	HR	95%Cl
**Gender**				
Female	ref			
Male	1.632	(0.908, 2.933)	1.634	(0.907, 2.943)
**Marital Status**				
Divorced	Ref			
Married	0.474	(0.321, 0.701)	0.479	(0.324, 0.708)
Single	0.404	(0.243, 0.671)	0.435	(0.261, 0.725)
Widowed	0.479	(0.303, 0.755)	0.474	(0.300, 0.749)
**Age**	1.001	(1.000, 1.013)	1.001	(1.001, 1.014)
**Grade**				
Undiff Anaplastic	Ref			
Grade I (Well diff)	0.227	(0.077, 0.673)	0.227	(0.076, 0.677)
Grade II (Mod diff)	0.281	(0.101, 0.779)	0.292	(0.104, 0.816)
Grade III (Poor diff)	0.453	(0.164, 1.254)	0.477	(0.171, 1.331)
**Topography**				
Nipple	Ref			
Central portion of breast	0.597	(0.340, 1.047)	0.620	(0.352, 1.089)
Upper-inner quadrant of breast	0.810	(0.491, 1.338)	0.849	(0.512, 1.407)
Lower-inner quadrant of breast	0.881	(0.509, 1.523)	0.913	(0.526, 1.584)
Upper-outer quadrant of breast	0.672	(0.446, 1.014)	0.682	(0.450, 1.031)
Lower-outer quadrant of breast	0.457	(0.260, 0.802)	0.472	(0.268, 0.830)
Axillary tail of breast	0.615	(0.211, 1.797)	0.625	(0.213, 1.830)
Overl. lesion of breast	0.781	(0.517, 1.179)	0.800	(0.528, 1.213)
TopographyC50.9 Breast, NOS	0.971	(0.656, 1.438)	1.042	(0.701, 1.548)
**Laterality**				
Bilateral Involve	ref			
Left	1.081	(0.602, 1.943)	1.027	(0.572, 1.846)
Paired site	4.413	(1.438, 13.546)	3.659	(1.177, 11.371)
Right	1.041	(0.579, 1.871)	0.997	(0.555, 1.791)

### Significance of the frailty term

Though variance for the frailty was small, the likelihood ratio test showed a significant heterogeneity. We tested the hypothesis under the likelihood ratio test that the estimate of the regional frailty was zero (*θ* = 0). Under this test, a chi-squared test statistic of 136.82 with a p-value < 0.001 was obtained. This result implies that *θ* was statistically significantly different from zero, suggesting an unobserved variation between or at regional levels, as well as other important or significant covariates that were unobserved.

### Kaplan-Meier survival curves for selected variables

**Fig 2:**
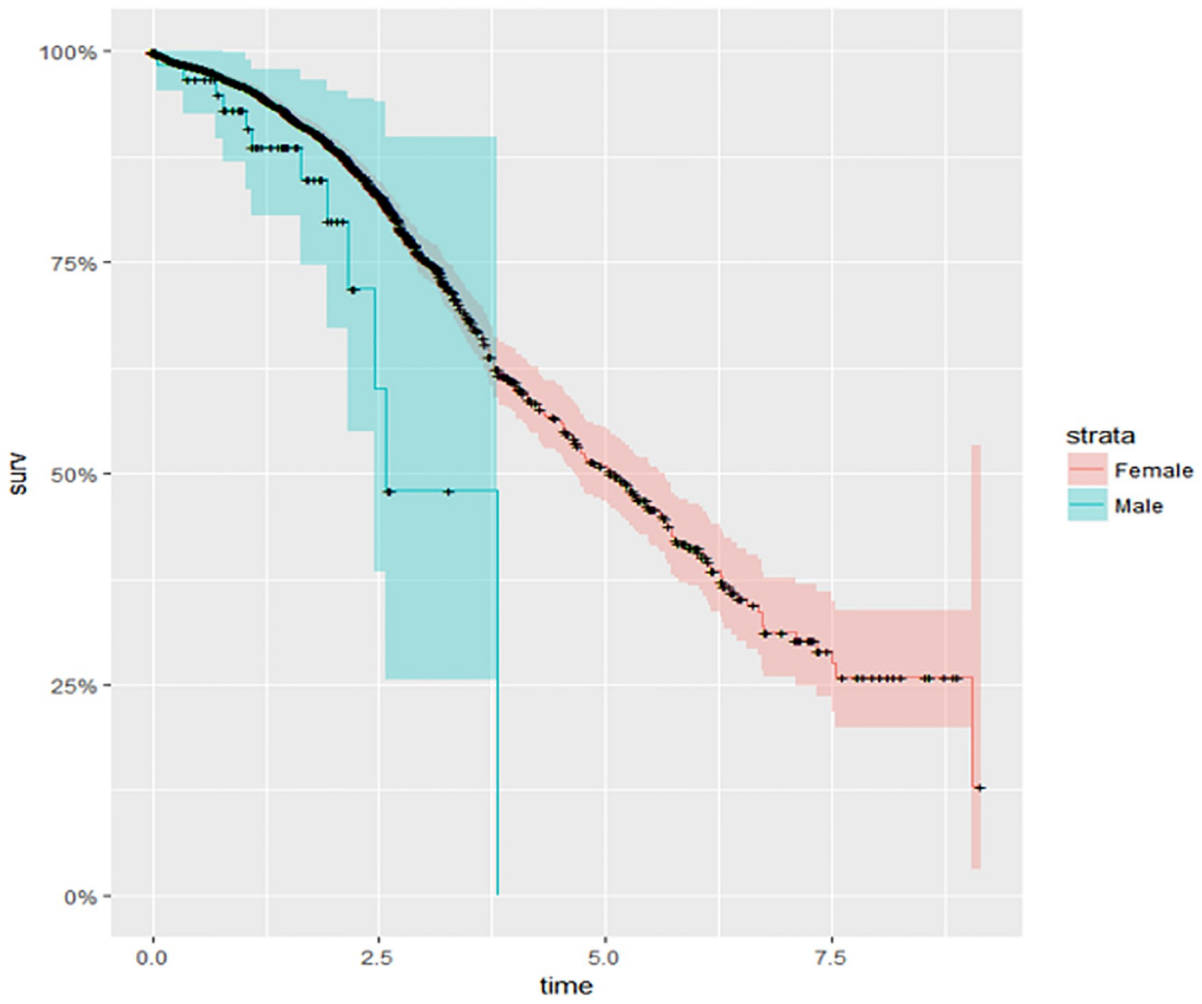
The Kaplan-Meier survival probability among men and women.

The Kaplan-Meier survival curves presented in Fig 2 indicate that survival among women with breast cancer in the Saudi Kingdom is higher than that for men. This observation may be due to lateness or lack of reporting among men. Because breast cancer is rare among men in this country and because it is regarded as a female-dominated disease, men may not report breast lumps or other related symptoms until the disease reaches later stages.

**Fig 3:**
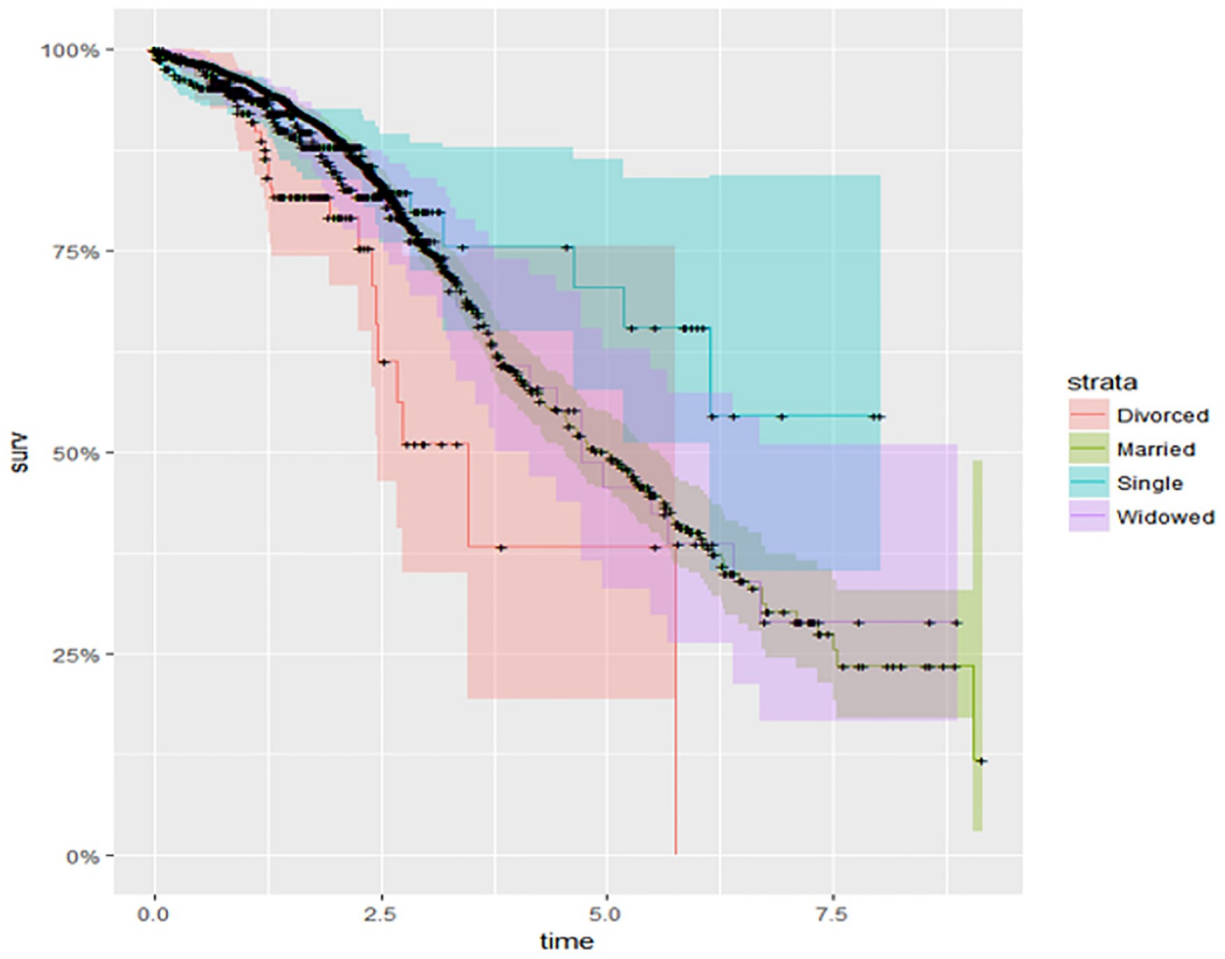
Kaplan-Meier survival probability on patients marital status.

**Fig 4:**
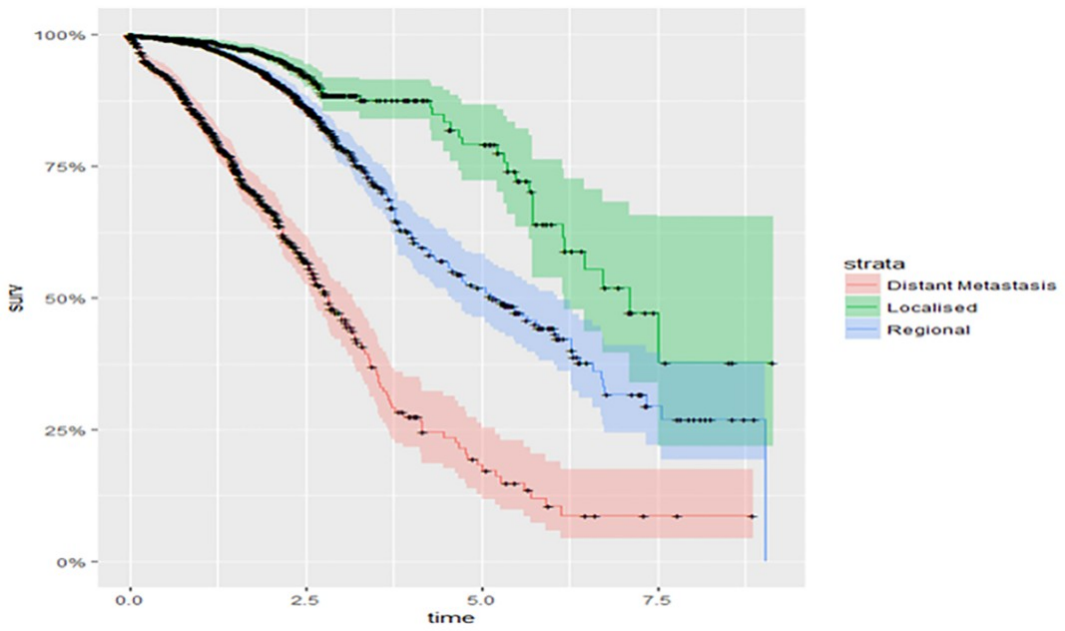
Kaplan-Meier survival probability on patients with extent or stage of the cancer.

The survival curves for marital status and the stage or extent of breast cancer as presented in Figs 3 and 4 show that single women have higher survival rates than other women. Divorced women have a higher hazard, or lower survival rate. Patients with localized cancers had lower risk of dying than those with regional and distant metastases, and those with regional cancers had higher survival than those with distant metastases. The global test for equality of survival functions indicated a statistically significant difference among all groups.

## Discussion

This paper investigated the determinants of breast cancer mortality among patients in the Kingdom of Saudi Arabia. Of the 708 deaths attributable to breast cancer in our sample, about 98% were women. Ito et al. [13] observed a similar higher mortality among women with breast cancer, mostly in Asian countries, which they attributed to changes in lifestyle caused by Western culture adaptation or influence. Prolonged introduction of efficient screening systems also may be a factor [13]. Hill et al. [14] found that mortality rates among women have stabilised or decreased in the last 25 years in the US. Efficient screening programmes and appropriate therapies could contribute to this trend [15].

Our analysis demonstrated that mortality among women was higher than that among men. Though mortality among men in Saudi Arabia and around the world are generally low, men who are diagnosed with breast cancer are more likely to die from the disease than women. According to the National Breast Cancer foundation, the higher prevalence of breast cancer among men may be attributed to lack of awareness, as men may not detect lumps in their breasts or report lumps to their health care providers and thus may receive delayed treatment for breast cancer. In line with our findings, Ly et al. [16] found that men were more at risk of dying from breast cancer than women, although this incidence varied according to country, with Israel having the highest and Thailand the lowest rates. Anderson et al. [17] observed a higher correlation of breast cancer between men and women. Their findings indicate some common risk factors between both sexes, unlike the findings reported by Muir et al. [18], who concluded that male breast cancers displayed immunophenotypic differences from female cancers. This finding suggests a difference in disease pathogenesis and progression that may warrant sex-specific treatments.

In this study, we found that in all cases, patients who were classified as divorced were twice as likely to die from breast cancer than their married counterparts. These findings are similar to those reported by Aizer et al. [19], who found that divorcees were about 21% more likely to die of breast cancer. A further analysis by Martinez et al. [20] similarly revealed that unmarried and widowed patients were 28% and 35% more at risk of death, respectively, compared to married ones. Their analysis was stratified according to cancer stage and is in accordance with our findings.

Gomez et al. [21] observed a stronger survival benefit among married people than unmarried ones, though men showed more benefits than women. They attributed these benefits to financial and social achievements. In examining the risk of marital status among women with breast cancer in Bangui, the capital city of the Central African Republic, Balekouzou et al. [22] showed that married women were more at risk of developing breast cancer than unmarried women. Other studies involving women from India and Iran support these conclusions [23], whereas some observed no relationship between marital status and breast cancer [24, 25]. Ross et al. [26] and Ballantyne [27] observed a protective effect against breast cancer among married women, which is similar to our findings showing that divorced women were twice and three times more likely to die from breast cancer than single and widowed women, respectively.

These finding implies that single and widowed women have more support, compared to their divorced counterparts. It also may be due to the cultural setting of the study population and their views on divorce. According to the Islamic religion, divorce is a vile act that must be avoided, because it jeopardises the throne of Allah [28]. This sentiment may account for the lack of support that divorced people receive, compared to others. Marriage also may promote healthy lifestyles and offer financial and psychological support. Although we did not conduct a separate analysis for men due to the small sample size, the results for women showed similar conclusions. According to a report by the Gazette Kingdom [29], the number of divorces in Saudi Arabia in 2017 was 40% to 45% out of 159,386 marriages, up from 27.86% in 2015.

We assessed four levels of lateral variability in tumours: bilateral, left, paired site, and right. We observed that patients with tumours on paired sites were about four times more at risk of dying than those with bilateral involvement. We also found that those with left-side tumours had a 49.85% chance of dying, compared to 47.74% for those with right-side tumours. Other studies have found that most tumours occur in the left breast [30, 31]. These findings may be attributable to several factors. First, mothers prefer to use their right breast during breastfeeding, though this preference may differ across regions [31]. Hartveit [31] also found that right-handed women check their left breast more often for lumps, increasing the chances of early treatment. A population-based case-control study found that women who are left-handed were at higher risk of developing breast cancer than right-handed women, though no statistical significance was established [32, 33].

## Conclusion

The descriptive analysis confirms a high prevalence of breast cancer mortality among Saudi Arabian women. Most of these deaths occurred in Riyadh, Makkah, and the eastern region of the Kingdom of Saudi Arabia. Though the prevalence of breast cancer was lower among men than among women, men were at more risk of death. Moreover, divorced patients were more at risk of death than married, single, and widowed patients, though there was high mortality among married patients. Saudi Arabian women with breast cancer who are divorced may have worse consequences and less support than other women because of the conservative nature of the country and its views about divorce.

We recommend an intensive health education programme for men and women that targets the consequences of divorce, especially considering the increasing divorce rate in the Kingdom of Saudi Arabia. Early diagnosis and treatment of breast cancer among men also are important steps in treating this disease. Some limitations should be noted. First, several observations (2901) with incomplete data were therefore dropped from the final analysis. If these number of observations were not dropped due to incompleteness, the results could have either been reinforced or changed. Second, the number of variables that were recorded and submitted to us for this work was not exhaustive.

## Appendices

### Appendix 1: The Cox proportional hazards model without a frailty term

The proportional hazards model specifies that the hazard at some time *t* for an individual with covariate *x* be written as

$$\begin{array}{c} h\left(t/x\right)=h_{0}\left(t\right)\text{exp}\left\{G\left(x,\beta \right)\right\} \end{array}$$ (1)

where *h*_0_(*t*) is the baseline hazard function with *β* being the vector of the regression coefficients. The exponential form exp[*G*(*x*, *β*)] is so expressed to ensure its positivity. Since the effect of covariates is multiplicative, the hazard function can be expressed as

$$\begin{array}{c} h\left(t|x\right)=h_{0}\left(t\right)\text{exp}\left(X^{\prime }\beta \right), \end{array}$$ (2)

where *X*′ represent the vector of covariates and *β* the regression coefficients.

The likelihood function (*h*_0_|(.), *β*) for the breast cancer (right censored) data on the *n* subjects is

$$\begin{array}{c} L(D|h_{0}\left(t\right),\beta )=\prod_{i=1}^{n}{\left\{h_{0}\left(t_{i}\right)\text{exp}\left(X_{i}^{\prime }\beta \right)\right\}}^{\delta_{i}}(S_{0}{\left(t_{i}\right)}^{\text{exp}(X_{i}^{\prime }\beta )}) \end{array}$$ (3)

### Appendix 2: The Cox proportional hazards model with a frailty term

Shared frailty model is the most common frailty models [34]. Assuming that the survival times for the *j*^*th*^ subject (*j* = 1, …, *m*) in the *i*^*th*^ group (*i* = 1, …, *n*) is denoted by *T*_*ij*_ with an unobserved frailty parameter given as *ω*_*i*_ (for the *i*^*th*^ group), then the hazard function can be written as

$$\begin{array}{c} \;h(t_{ij}|X_{ij},\omega_{i})=h_{0}\left(t\right)\text{exp}(\sigma \omega_{i}+X_{ij}\beta ) \end{array}$$ (4)

where *ω*_1_, …, *ω*_*n*_ represent the frailty and *h*_0_(*t*), *X*_*ij*_ and *β* hold same as expressed previously. We assume that the frailties (*ω*’s) are independently sampled from a distribution with mean 0 and variance *σ*. This implies that if *σ* is zero, then Eq 4 will reduce to the standard Cox proportional hazards model.

In some situations, it is more appropriate to rewrite the model in Eq 4 as

$$\begin{array}{c} h(t_{ij}|X_{ij},u_{i})=h_{0}\left(t_{ij}\right)\text{exp}(X_{ij}^{\prime }\beta )u_{i} \end{array}$$ (5)

where the *u*_*i*_’s are independently and identically distributed from a distribution with mean 1 and variance *θ*.

The frailty distribution for each of *u*_*i*_ is assumed to be independent gamma following Clayton [35] and expressed as

$$\begin{array}{c} u_{i}∼Gamma(\eta ,\eta ),i=1,\ldots ,n \end{array}$$ (6)

where *η*^−1^ is the unknown variance of *u*_*i*_. We assume that

$$\begin{array}{c} X∼Gamma\left(a,b\right)\propto x^{a-1}\text{exp}\left(-bx\right),\;\text{for}\;x>0,a>0\;\text{and}\;b>0. \end{array}$$

### Appendix 3: Bayesian proportional hazards model with/without a frailty term

The posterior probability density function which summarises our beliefs about a particular parameter is obtained via the Bayes’ rule as

$$\begin{array}{c} \pi \left(\theta |D\right)=\frac{\pi (\theta )L(D|\theta )}{\int_{\Theta }\pi (\theta )L(D|\theta )d\theta } \end{array}$$ (7)

Which can be summarised as

$$\begin{array}{c} \pi (\theta |D)\propto \pi (\theta )L(D|\theta ) \end{array}$$ (8)

With this approach, the ∝ hides the marginalised constant ∫_Θ_ *π*(*θ*)*L*(*D*|*θ*)*dθ* which does not depend on the parameter *θ*. Therefore, the posterior distribution can be obtained as

$$\begin{array}{c} \pi (h_{0}\left(t\right),\beta |D)\propto \prod_{i=1}^{n}{\left\{h_{0}\left(t_{i}\right)\text{exp}\left(X_{i}^{\prime }\beta \right)\right\}}^{\delta_{i}}(S_{0}{\left(t_{i}\right)}^{\text{exp}(X_{i}^{\prime }\beta )})\pi \left(\beta \right) \end{array}$$ (9)

where the prior distribution for the regression coefficients *β* are assigned normal distributions with mean *μ*_0_ and variance $\sigma_{0}^{2}$ with a probability density function given as

$$\begin{array}{c} f\left(x|\mu_{0},\sigma_{0}^{2}\right)=\frac{1}{\sqrt{2\pi }\sigma_{0}^{2}}e-\frac{{\left(x-\mu_{0}\right)}^{2}}{2\mu_{0}^{2}}. \end{array}$$ (10)

In analysing the frailty parameter (*u*) via the Bayesian approach, we adopt a conjugate prior for the hyperparameters *η*, a Gamma distribution with a constant mean and some large variance.
